# Cardiorespiratory and Neuromuscular Demand of Daily Centrifugation: Results From the 60-Day AGBRESA Bed Rest Study

**DOI:** 10.3389/fphys.2020.562377

**Published:** 2020-09-11

**Authors:** Andreas Kramer, María Venegas-Carro, Edwin Mulder, Jessica K. Lee, María Moreno-Villanueva, Alexander Bürkle, Markus Gruber

**Affiliations:** ^1^Department of Sport Science, University of Konstanz, Konstanz, Germany; ^2^Institute of Aerospace Medicine, German Aerospace Center (DLR), Cologne, Germany; ^3^Department of Biology, University of Konstanz, Konstanz, Germany

**Keywords:** exercise, countermeasure, heart rate, oxygen consumption, artificial gravity

## Abstract

**Purpose:**

Long stays in space require countermeasures for the degrading effects of weightlessness on the human body, and artificial gravity (AG) has been proposed as an integrated countermeasure. The aim of this study was to assess the cardiorespiratory and neuromuscular demand of AG elicited via daily centrifugation during 60 days of bed rest.

**Methods:**

Twenty four participants (33 ± 9 y, 175 ± 9 cm, 74 ± 10 kg, 8 female) were subjected to 60 days of strict six-degree head-down tilt (HDT) bed rest and were randomly allocated to one of three experimental groups: 30 min of daily centrifugation with an acceleration of 1 g at the center of mass and 2 g at the feet applied continuously (cAG) or intermittently in 6 epochs of 5 min each, separated by 3 min breaks (iAG), or non-centrifuged control (CTRL). Cardiorespiratory demand during centrifugation was assessed at the beginning (HDT3) and end (HDT60) of the bed rest phase via spirometry and heart rate monitoring, leg muscle activation was monitored via electromyography.

**Results:**

On average, analyses of variance revealed that heart rate during centrifugation increased by 40% (iAG) and 60% (cAG) compared to resting values (*p* < 0.001), while oxygen uptake did not change significantly (*p* = 0.96). There was a preference for calf over knee extensor muscle activation (active time soleus 57 ± 27%, gastrocnemius medialis 45 ± 27% and vastus lateralis 27 ± 27%, *p* < 0.001), with large inter-individual differences in leg muscle active time. AG could not prevent the increase in resting heart rate after bed rest. For most of the recorded parameters, there were little differences between cAG and iAG, with the increase in heart rate during centrifugation being a notable exception (greater increase for cAG, *p* = 0.01).

**Conclusion:**

Daily 30 min bouts of artificial gravity elicited by centrifugation put a substantial demand on the heart as a pump without increasing oxygen consumption. If centrifugation is to be used as a countermeasure for the deteriorating effects of microgravity on physical performance, we recommend combining it with strenuous exercise.

## Introduction

The deconditioning of astronauts during long-duration space missions is a well-recognized risk (Williams et al., 2009). The absence of gravitational loading is associated with reduced physical performance, similar to what can be observed with physical inactivity or physiological aging on Earth. In addition, the cranial fluid shift (i.e., primarily from the legs toward torso and head) and the loss of plasma volume have been associated with effects on orthostatic tolerance and the development of the Spaceflight-Associated Neuro-Ocular Syndrome (SANS).

To counteract spaceflight-associated adaptations, a variety of countermeasures have been developed and tested both in space and in space analog models such as bed rest with head-down tilt to mimic the cranial fluid shift (Hargens et al., 2013; Kozlovskaya et al., 2015). For the deterioration of physical performance associated with gravitational unloading and physical inactivity, an effective countermeasure has already been established during a previous bed rest study: in the 60 day bed rest study, a short intensive jump training program consisting of countermovement jumps and repetitive hops was tested and was able to prevent the large musculoskeletal and cardiovascular deconditioning effects, in particular the loss of bone mineral mass and density, lean muscle mass, maximal leg strength and power as well as peak oxygen uptake (Kramer et al., 2017a, b, 2018). However, the effects associated with plasma volume loss and fluid shift have not yet been addressed satisfactorily. One countermeasure that has the potential to address all of these effects is artificial gravity: as most problems of astronaut health are the result of lacking gravitational force, substituting this gravitational force with some kind of artificial gravity, for instance via centrifugation in a short-arm centrifuge, could alleviate many of these problems. Indeed, in a recent review Clement et al. (2015) summarized the findings of short-arm centrifugation during bed rest and reported improved orthostatic tolerance time, attenuated plasma volume loss, higher exercise capacity and less severe responses to head-up tilt, especially if the centrifugation is administered in an intermittent fashion (Linnarsson et al., 2015). In addition to the expected benefits for cardiovascular function, it has been suggested that short-arm centrifugation could also help to prevent muscle atrophy, bone demineralization, and impairment of neuromuscular and sensorimotor coordination (Linnarsson et al., 2015). However, these studies were too short in duration to thoroughly study the efficacy of short-arm centrifugation on neuromuscular function and bone health.

In the bed rest study described in this paper – organized by the European Space Agency (ESA), the National Aeronautics and Space Administration (NASA), and the German Aerospace Center (DLR), and hosted by the DLR’s Institute of Aerospace Medicine – several teams of researchers were involved, using the study as an opportunity to study among others cardiovascular, cognitive and neuromuscular adaptations to bed rest. The main goal of the study (named AGBRESA, short for “Artificial Gravity Bed Rest ESA”) was to assess the efficacy of daily centrifugation in preventing the deconditioning of the human body during 60 days of bed rest. In this manuscript, we quantified the cardiorespiratory and neuromuscular demand during the daily 30 min-bouts of centrifugation, and analyzed whether any changes occurred over the course of the bed rest phase. We hypothesized that acute centrifugation would increase heart rate and oxygen uptake, and little difference between the two intervention groups.

## Materials and Methods

### Study Design

This randomized controlled study was conducted in 2019 at the:envihab facility of the German Aerospace Center (DLR) in Cologne, Germany. The study was split into two campaigns with 12 participants each. Each campaign consisted of 14 days of baseline data collection (BDC-14 through BDC-1), 60 days of head-down tilt bed rest (HDT1 through HDT60), and 14 days of recovery (R + 0 through R + 13). Data for the part of the study described in this paper were recorded on days HDT3 and HDT60.

### Subjects

At the end of the second campaign, 24 participants had successfully completed the study. In the morning of HDT1, subjects were randomly assigned to either the intermittent centrifugation group (iAG, *n* = 8, age 34 ± 11 years, height 174 ± 11 cm, body mass 71 ± 5 kg, 3 females), the continuous centrifugation group (cAG, *n* = 8, age 32 ± 10 years, height 173 ± 8 cm, body mass 72 ± 10 kg, 3 females) or the control group (CTRL, *n* = 8, age 34 ± 8 years, height 177 ± 7 cm, weight 79 ± 13 kg, 2 females). Before taking part in the study, all participants gave written informed consent to the experimental procedures, which were approved by the ethics committee of the Northern Rhine Medical Association (Ärztekammer Nordrhein, application #2018143) in Duesseldorf, Germany, as well as the Federal Office for Radiation Protection (Bundesamt für Strahlenschutz). Subjects received a monetary compensation for participating in the study. The study is registered at the German Clinical Trials Register (DRKS00015677).

### Bed Rest Routine

During the bed rest period (starting at 9 a.m. of HDT1), the subjects maintained strict 6° HDT bed rest for 24 h/day. All activities, including personal hygiene activities (bowel movement, showering etc.) were performed in the HDT position. Previous studies identified that the usage of a pillow might confound results (Lawley et al., 2017; Laurie et al., 2019). Therefore, the use of a pillow was prohibited once the subject entered the HDT phase of the study. Round-the-clock staff and video monitoring ensured compliance with the protocol. During the baseline and recovery phases (BDC and R), physical activity was restricted to free movement within the ward as well as reconditioning sessions administered by physiotherapists. During the entire study, the subjects received a strictly controlled diet, with a water intake of 50ml/kg body mass and an energy intake of 1.6 times the resting metabolic rate during the ambulatory phases (BDC and R) and 1.3 times the resting metabolic rate during HDT. Resting heart rate was measured every morning in fasting state immediately after waking up (Intellivue MMS X2; Philips, Best, The Netherlands).

### Centrifugation

**FIGURE 1:**
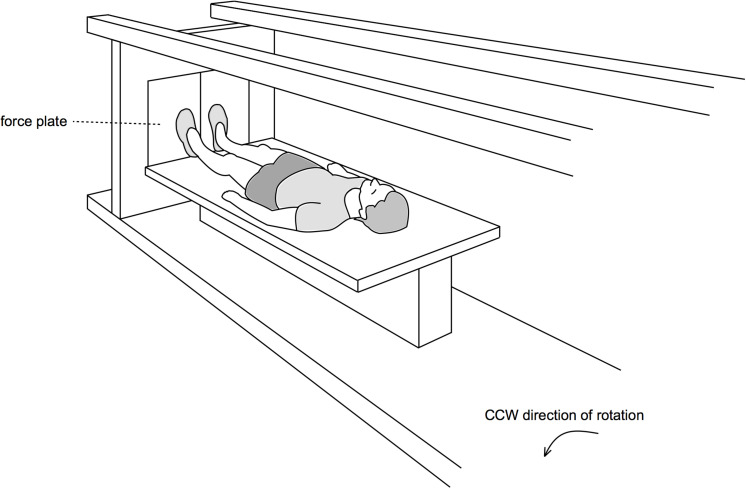
Centrifuge. For the artificial gravity intervention, a short-arm human centrifuge was used. Participants lay supine on a thin mattress on one arm of the centrifuge, feet pointing outwards. Rotational speed and the participant’s position with respect to the center of rotation were individually adjusted to achieve an acceleration of 1g at the estimated center of mass and approximately 2 g at the feet.

The DLR short-arm human centrifuge has a radius of 3.8 m, for a schematic (see Figure 1), for more details see previous publications using this centrifuge (Kramer et al., 2020). The participant’s position with respect to the center of rotation as well as the rotational speed of the centrifuge was adjusted to achieve an acceleration of 1 g at the estimated center of mass and 2 g at the feet. All subjects were familiarized with the centrifugation twice during the baseline phase (BDC-11 and BDC-4). Participants were instructed to stay in a supine position during centrifugation, gaze fixed on a point, avoid head movements and only contract the leg muscles to avoid presyncopal symptoms. Participants in the two intervention groups (cAG and iAG) completed one daily session of 30 min of centrifugation, continuous for cAG and interspersed with 3 min breaks in between the 5 min bouts of centrifugation for iAG. These centrifugation protocols were based on the protocols used in a previous short-term bed rest study (Rittweger et al., 2015) and aimed to assess benefits of and differences between the two protocols with respect to AG tolerance and SANS. All sessions were supervised by a medical doctor and were carried out in a horizontal position, i.e., at 0° HDT. Prior to, and after the end of the centrifuge run, subjects were positioned in 6° HDT, whilst on the centrifuge. Rotation direction was changed from day to day so that every participant was scheduled for 30 sessions with clockwise direction of rotation and 30 sessions with counter-clockwise rotation. Rotation direction per participant on HDT3 and HDT60 was kept constant to ensure comparability (half of the participants in cAG were spun clockwise on both days, half of them counter-clockwise, same for iAG).

### Spirometry and Heart Rate Monitoring

**FIGURE 2:**
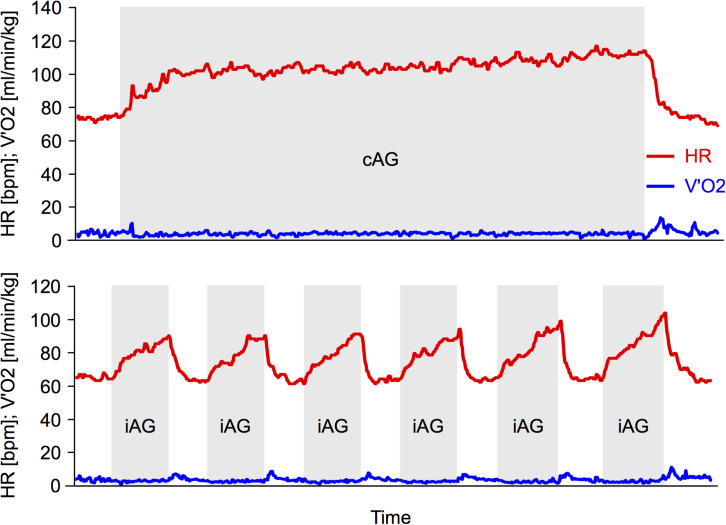
Exemplary data during centrifugation. **Top:** exemplary data from one session from one participant of the continuous artificial gravity group (cAG). **Bottom:** exemplary data from one session from one participant of the intermittent artificial gravity group (iAG). Red lines represent the heart rate (HR), blue lines the oxygen consumption (V’O_2_). The cAG consists of 30 min of continuous centrifugation, whereas the iAG consists of 6 × 5 min of centrifugation (marked in gray), interspersed with breaks of 3 min.

Breath-by-breath oxygen uptake and carbon dioxide emission before and during the HDT3 and HDT60 artificial gravity sessions were monitored using the Cortex system (Cortex Metalyzer, Germany). Heart rate was continuously monitored via a chest belt (Polar Pro, Finland). The spirometry data was filtered by taking the median of 5 breaths. A baseline recording was taken just prior to the centrifuge run (average of the 3 min before centrifuge start for both oxygen uptake and heart rate). To assess the cardiorespiratory demand during centrifugation, the average and maximum values during the 30 min of centrifugation were taken (excluding the 3 min breaks as well as the few seconds of spin-up and spin-down for iAG). For exemplary data of two participants (cAG and iAG, respectively) (see Figure 2).

### Electromyography

Wireless surface electrodes (Noraxon Ultium, United States) were placed over M. soleus (SOL), M. gastrocnemius medialis (GM) and M. vastus lateralis (VL) of the right and left leg. The longitudinal axes of the electrodes were in line with the presumed direction of the underlying muscle fibers. Interelectrode resistance was reduced by shaving and light abrasion of the skin. The EMG signals were wirelessly transmitted to the base station (band-pass filter 20–500 Hz) and sampled with 2 kHz. After removing DC offsets, the EMG signals were rectified. Baseline EMG activity for each participant in cAG and iAG was calculated as the mean of a 10 s-interval at the beginning of the centrifuge run, when the centrifuge was at its target rotational speed and the participant was not using the muscle pump. This value was used to calculate each muscle’s active time during centrifugation, i.e., the percentage of the time spent above baseline EMG activity plus three standard deviations. As the groups were counterbalanced for rotation direction and there were no significant differences between the left and the right leg, the average of both legs was used.

### Statistics

Group data are presented as means ± standard deviations (SD). The analyses were executed using JASP 0.84 (University of Amsterdam, Netherlands). To assess whether the demand of the two artificial gravity interventions changed from the beginning of bed rest to the end of bed rest, repeated measures analyses of variance (rmANOVA) were used, with time (HDT3 and HDT60) as repeated measure and group (cAG, iAG) as inter-subject factor. The same analyses were used for resting heart rate, but with three groups (cAG, iAG, CTRL). The demand of centrifugation (V’O_2_, HR) compared to baseline levels at rest was also assessed with rmANOVAs, using centrifugation (baseline and centrifugation) as repeated measure and group (cAG, iAG) as inter-subject factor. Last, muscle activity during centrifugation was additionally analyzed with ANOVAs, using muscle (SOL, GM, VL) as within-subject factor and group (cAG, iAG) as inter-subject factor. Significance level was set to 0.05.

## Results

### Heart Rate

**FIGURE 3:**
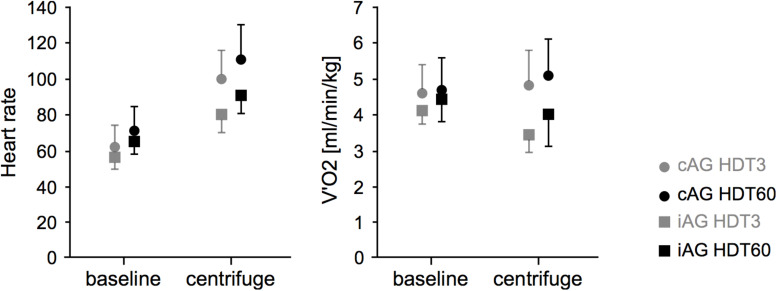
Mean and standard deviation of heart rate (HR) and oxygen uptake (V’O_2_) during the two artificial gravity session (HDT3 in gray, HDT60 in black) for the two intervention groups (cAG is represented by circles, iAG by squares). Baseline values were recorded and averaged over the 3 min before the start of the centrifuge run, centrifugation values over the 30 min of centrifugation.

**FIGURE 4:**
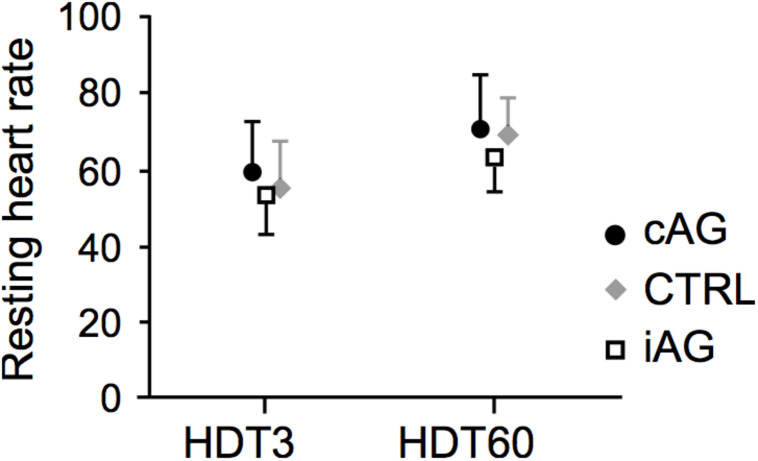
Resting heart rate at the beginning (HDT3) and the end (HDT60) of the bed rest phase (cAG is represented by filled circles, CTRL by gray diamonds and iAG by open squares). There was a similar increase in resting heart rate for all three groups (no time ^∗^group interaction effect, *p* = 0.39; significant main effect of time, *p* < 0.001).

For cAG, average heart rate during centrifugation was about 60% higher compared to baseline values, whereas for iAG it increased by 40%, with a significant centrifugation^∗^group interaction effect (*p* = 0.003 for HDT3 and *p* = 0.01 for HDT60) and a significant main effect of centrifugation (*p* < 0.001) (see also Table 1 and Figure 3). The increase in heart rate during centrifugation compared to baseline levels did not change from HDT3 to HDT60 (*p* = 0.3, see Table 1). There was also a significant main effect of time, i.e., an increase in heart rate from the beginning of bed rest (HDT3) to the end of the bed rest (HDT60). This increase was observed both in the resting heart rate measured in the morning before breakfast (see Figure 4; CTRL 57 ± 10 on HDT3 vs. 68 ± 8 on HDT60, cAG and iAG see Table 1), and in the values before and during centrifugation (baseline, average and maximum, see Table 1).

**TABLE 1:** Means and standard deviations of heart rate (HR), oxygen uptake (V’O_2_) and muscle activity (EMG) for the two intervention groups (cAG and iAG) during the artificial gravity sessions on the third (HDT3) and last (HDT60) day of head-down tilt bed rest.

	**cAG HDT3**	**cAG HDT60**	**iAG HDT3**	**iAG HDT60**	Time*Group	**Main effect of time**	**Main effect of group**
Resting HR	59 ± 14	69 ± 13	55 ± 12	62 ± 7	*p* = 0.39	*p* < *0.001*	*p* = 0.39
HR baseline	63 ± 11	71 ± 14	58 ± 7	65 ± 8	*p* = 0.93	*p* < *0.001*	*p* = 0.35
HR centrifuge	100 ± 15	111 ± 20	80 ± 10	91 ± 10	*p* = 0.93	*p* = *0.01*	*p* = *0.02*
	159 ± 15%	156 ± 17%	140 ± 7%	140 ± 12%	*p* = 0.92	*p* = 0.30	*p* = *0.01*
	[138–177]	[131–179]	[129–148]	[123–158]			
HR max	115 ± 12	130 ± 22	96 ± 14	111 ± 13	*p* = 0.92	*p* = *0.01*	*p* = *0.02*
V’O_2_ baseline [ml/min/kg]	4.6 ± 0.7	4.7 ± 0.9	4.1 ± 0.3	4.4 ± 0.6	*p* = 0.24	*p* = 0.62	*p* = 0.28
V’O_2_ centrifuge [ml/min/kg]	4.8 ± 1.0	5.1 ± 1.1	3.5 ± 0.5	4.0 ± 0.8	*p* = 0.61	*p* = 0.15	*p* = *0.01*
	106 ± 18%	111 ± 23%	86 ± 9%	90 ± 9%	*p* = 0.64	*p* = *0.03*	*p* = *0.02*
	[85–135]	[85–164]	[71–93]	[79–103]			
EMG SOL active [% of total]	52 ± 35	60 ± 31	56 ± 29	54 ± 25	*p* = 0.64	*p* = 0.82	*p* = 0.97
	[9–92]	[19–96]	[1–85]	[21–80]			
EMG GM active [% of total]	37 ± 33	45 ± 27	29 ± 19	45 ± 29	*p* = 0.58	*p* = 0.26	*p* = 0.84
	[0–78]	[4–80]	[1–54]	[2–84]			
EMG VL active [% of total]	30 ± 33	39 ± 34	11 ± 11	15 ± 13	*p* = 0.96	*p* = 0.36	*p* = 0.17
	[1–81]	[3–85]	[0–26]	[2–33]			

**Values in brackets refer to the range across participants. Resting HR was measured in the morning before breakfast; HR baseline and V’O_2_ baseline refer to the average values of the 3 min before the start of the centrifuge run; HR centrifuge and V’O_2_ centrifuge refer to the average values of the 30 min of centrifugation (percentages refer to the ratio of the centrifuge values with respect to baseline levels, i.e., 159% means that the average HR during centrifugation was 59% higher than baseline HR); HR max refers to the maximal values of the 30 min of centrifugation; Muscle activity of the three recorded leg muscles SOL, soleus; GM, gastrocnemius medialis; VL, vastus lateralis is expressed as active time, i.e., the percentage of the 30 min of centrifugation that the muscle activity was higher than baseline activity. The p-values are given for the ANOVAs comparing the two AG interventions over time; for the results of the analyses comparing the demand of centrifugation to baseline levels, see text body of the respective results subsection.**

### Oxygen Uptake

Average oxygen uptake during centrifugation was not significantly different from oxygen uptake at rest: it increased non-significantly by about 10% for cAG and decreased by about 10% for iAG (see Table 1 and Figure 3). Normalized to the maximal oxygen uptake V’O_2__max_, this translates to a change from a baseline level of 13 ± 4% to a level of 14 ± 4% during centrifugation for cAG, and from a baseline level of 13 ± 3% to a level of 12 ± 3% during centrifugation for iAG (V’O_2__max_ data recorded on BDC-3 during cycling spiroergometry, provided by ESA for normalization purposes). The centrifugation^∗^group interaction effect was *p* = 0.04 for HDT3 and *p* = 0.06 for HDT60, with non-significant main effects of centrifugation (*p* = 0.33 for HDT3 and *p* = 0.95 for HDT60).

### Electromyography

**FIGURE 5:**
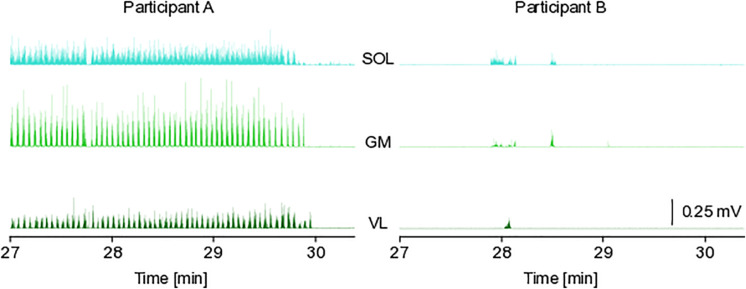
Exemplary EMG data. The figure depicts exemplary electromyographic data from two participants (both cAG to facilitate the comparison) during the last 3 min of their 30 min centrifugation session. The data was recorded from the SOL, soleus; GM, gastrocnemius medialis; VL, vastus lateralis muscles. Participant A was almost constantly active, whereas participant B rarely contracted the leg muscles.

**FIGURE 6:**
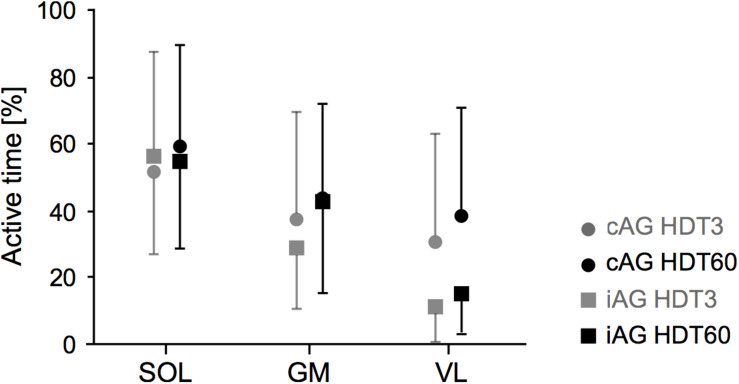
EMG activity during centrifugation. Mean and standard deviation of the active time (percentage spent above baseline EMG activity level) of the three recorded leg muscles (SOL, soleus; GM, gastrocnemius medialis; VL, vastus lateralis) during the two artificial gravity session (HDT3 in gray, HDT60 in black) for the two intervention groups (cAG is represented by circles, iAG by squares).

The analysis of the muscle activity recordings during centrifugation revealed large inter-individual differences, with some participants contracting their leg muscles almost continuously, and some participants barely contracting the leg muscles during the entire 30 min of centrifugation, resulting in a range between below 1 and 96% muscle active time during centrifugation (see Table 1 and Figure 5). There were no group^∗^time, time or group effects (see Table 1), but there was a clear difference between muscles (main effect of muscle for the muscle × group ANOVA, *p* < 0.001), with muscle active time increasing from VL (about 15% for iAG) to GM (about 35%) to SOL (about 55%) (see Figure 6 and Table 1). Note that especially for VL the standard deviations of the cAG group are quite high due to two participants who almost constantly used the muscle pump to avoid presyncopal symptoms.

## Discussion

Centrifugation increased heart rate by about 50%, but did not significantly change oxygen uptake. There were large inter-individual differences in leg muscle activation, and plantar flexor muscles were used more than knee extensors. Differences between the two intervention groups were not pronounced, but the cAG group did exhibit a greater increase in heart rate and a slightly higher oxygen uptake and muscle activity compared to iAG.

The observation that centrifugation substantially increased heart rate above baseline levels without a concomitant increase in oxygen consumption may seem surprising at first glance, especially in light of the close relationship between heart rate and oxygen uptake observed in common exercise physiology tests (Hossack, 1987; Weston and Gabbett, 2001). In these tests and during aerobic exercise in general, heart rate increases to meet the oxygen and energy demand of the working muscles. During centrifugation however, the increase in heart rate serves to compensate the decrease in stroke volume, cardiac output and venous return, to counteract the effects of the centrifugal forces that force blood into the distal body parts, notably the legs (Vettes et al., 1980; Pendergast et al., 2012).

The increase in heart rate from HDT3 to HDT60 that could be observed for all measurements – resting heart rate in the morning, baseline before centrifugation, average and maximal heart rates during centrifugation – was similar in all respective groups. This indicates that artificial gravity was not successful in completely preventing cardiovascular deconditioning, as similar increases in resting heart rate have been reported in response to bed rest (Spaak et al., 2005; Shi et al., 2014), but could be prevented by intensive exercise in a previous 60 day bed rest study (Kramer et al., 2017b). Of course, resting heart rate alone is not enough to assess the effects of the centrifugation on cardiovascular health. However, other data such as V’O_2__max_ data support this notion and will be published by other research groups.

The fact that oxygen uptake was not significantly increased during centrifugation shows that centrifugation alone should not be regarded as exercise, or as very light exercise at most when repetitively contracting the leg muscles to support venous return. Even the participants that used the muscle pump almost continuously had an increase in oxygen uptake of only 64% compared to supine baseline values (see ranges in Table 1), which translates to a normalized increase from 12% of V’O_2__max_ at baseline to 20% of V’O_2__max_ during centrifugation. For comparison’s sake: the American College of Sports Medicine’s position stand classifies exercise below 37% of V’O_2__max_ as “very light” (Garber et al., 2011). This is similar to the results from studies comparing oxygen uptake during supine and upright body positions that report only minor differences (Mcgregor et al., 1961; Leyk et al., 1994; Koga et al., 1999), and supports the results of previous studies proposing that cardiovascular responses to centrifugation with 2 g at foot level can be compared to those observed during standing (Goswami et al., 2015; Verma et al., 2018). Exercise during centrifugation in a supine position has even been associated with lower oxygen uptake compared to the same exercise in an upright position without centrifugation, presumably because the whole body is supported during centrifugation, eliminating the need for constant postural adjustments (Piotrowski et al., 2018). Studies comparing exercise in a recumbent position with and without centrifugation found small increases in cardiovascular response with higher artificial gravity levels (Diaz-Artiles et al., 2018), whereas passive centrifugation has been associated with reduced cardiac output, reduced pulmonary perfusion capacity and thus reduced oxygen transport (Pendergast et al., 2012). In contrast to the low oxygen demand observed during centrifugation, exercise countermeasures that have been tested in bed rest studies with similar duration had much higher cardiorespiratory demands. For instance, the high-intensity jump training employed in the RSL study has been shown to elicit peak heart rate and oxygen uptake values close to the maximal values recorded in a V’O_2__max_ test (Kramer et al., 2019).

The analysis of the leg muscle activity revealed that (a) the calf muscles were more active than the thigh muscles and that (b) there were large inter-individual differences with respect to the duration and intensity of the muscle contractions, i.e., some participants hardly contracted their leg muscles during centrifugation beyond the normal background activity, whereas others were almost continuously using the muscle pump to avoid presyncopal symptoms. Notably, the two participants who almost continuously used the muscle pump during the 30 min of centrifugation were the only participants to exhibit a pronounced increase in oxygen consumption above baseline levels. Both of these participants were in the cAG group, which at least partly explains the small differences observed between cAG and iAG (average oxygen consumption slightly increasing during centrifugation for cAG and slightly decreasing for iAG). Thus, the differences between iAG and cAG with respect to oxygen consumption and EMG activity are potentially driven by outliers, and should therefore be interpreted with caution. Note that excluding these two participants from the analyses would make the means of the two groups very similar, but the ranges would still be quite wide, see ranges for the “non-affected” iAG group reported in Table 1. Data from the medical monitoring of the participants during centrifugation suggests that this was not due to a lack of participant compliance, but due to inter-individual differences in artificial gravity tolerance. Thus, standardization of “passive” centrifugation seems to be inherently difficult, but could be remedied by adding adequate exercise, leading to similar muscle activation for all participants.

## Limitations

As it is usually the case with bed rest or astronaut studies, the sample size in the present bed rest study was substantial for such a type of study, but still rather small. Combined with large inter-individual differences, this limits the analysis of subtle group differences, as the inter-individual differences were sometimes larger than the between-group differences. As the main objective of the present study was to quantify the cardiorespiratory and neuromuscular demand of centrifugation during bed rest (and not primarily to analyze small differences between the two groups), this limitation can be considered a minor issue.

The analyses of muscle activity would have benefited from a recording of muscle activity during a maximal voluntary contraction, thus allowing a normalization of muscle activity during centrifugation. However, maximal muscle contractions during bed rest were not allowed due to potential confounding effects: for instance, during a previous 56 day bed rest study, maximal strength tests during bed rest resulted in a less pronounced decrease in muscle strength (Mulder et al., 2006). In the current study, the analyses of the non-normalized EMG activity confirmed the results of the muscle active times: participants with longer active times during centrifugation usually also showed higher average muscle activity levels, i.e., they not only contracted their muscles longer but also stronger. However, due to a number of issues that arise when using non-normalized EMG data (notably the difficulty in comparing data across subjects, muscles and time points), we did not include these analyses in this manuscript.

## Conclusion

Daily 30 min bouts of artificial gravity elicited by centrifugation put some demand on the heart as a pump, but did not translate to an increase in oxygen consumption. There were large inter-individual differences regarding the use of the muscle pump, but even the participants who rhythmically contracted their muscles during the whole artificial gravity session only increased their oxygen uptake by a small amount. Also, artificial gravity could not prevent the increase in resting heart rate observed after the 60 days of bed rest. Thus, centrifugation should be combined with strenuous exercise to potentially become an integrated countermeasure for the deteriorating effects of microgravity.

## Data Availability Statement

The raw data supporting the conclusions of this article will be made available by the authors, without undue reservation, to any qualified researcher.

## Ethics Statement

The studies involving human participants were reviewed and approved by the Northern Rhine Medical Association (Ärztekammer Nordrhein, application #2018143) in Duesseldorf, Germany. The patients/participants provided their written informed consent to participate in this study.

## Author Contributions

AK wrote the grant proposal, designed the study, acquired and analyzed the data, and drafted the manuscript. MV-C acquired data and revised the manuscript. EM, JL, MM-V, and MG contributed to the study preparation and revised the manuscript. All authors approved the final manuscript and are accountable for all aspects of the work.

## Conflict of Interest

The authors declare that the research was conducted in the absence of any commercial or financial relationships that could be construed as a potential conflict of interest.
